# Fiber Bragg Grating Based Load Monitoring for Carrier-Based Aircraft Main Landing Gear

**DOI:** 10.3390/s25175559

**Published:** 2025-09-05

**Authors:** Weijun Xue, Heng Huang, Xiwen Pang, Guang Yan

**Affiliations:** 1 College of Aerospace Engineering, Nanjing University of Aeronautics and Astronautics, Nanjing 210016, China; xueweijun2025@163.com; 2 College of Instrument Science and Opto-Electronics Engineering, Beijing Information Science & Technology University, Beijing 100101, Chinapangxw4977@163.com (X.P.)

**Keywords:** landing gear, fiber grating, strain, calibration equation, load regression

## Abstract

A three-dimensional load regression system based on fiber Bragg grating strain sensor is proposed to meet the load monitoring requirements of the main landing gear of an aircraft during take-off and landing. The FBG sensors, featuring a strain resolution of 1 με and a strain sensitivity of 1.18 pm/με, were selected to ensure precise strain acquisition. Through three-dimensional modeling and static simulation of the main landing gear, the strain response trend of the structure under this load state is obtained as a reference for sensor placement. On this basis, the sensor networking scheme is designed, and the ground static load of the main landing gear is calibrated. The strain–load regression matrix model for the measured main landing gear is constructed through the collected strain data, and the reliability of its prediction is verified. The results show that the system can effectively monitor the structural load, and the error between the back-calculated regression load and the applied load is within 4%.

## 1. Introduction

Landing gear, as the key load-bearing structure of aircraft, plays an important role in buffering during takeoff and landing. However, the landing gear works in a complex environment and bears a variety of alternating loads, and it is also the subsystem that is most prone to failure in air transportation [1]. The rapid development of the aviation industry also puts forward more requirements for the safety and reliability of landing gear. In order to ensure flight safety, it is particularly important to carry out real-time and online structural monitoring of landing gear structures [2,3].

With the breakthrough of nondestructive testing, sensor technology and data analysis, the health monitoring technology of landing gear structure has developed rapidly. The traditional electric sensing measurement method is simple in installation and intuitive in data display, and has been widely used in the load monitoring of landing gear. For example, Michael [4] of the American Institute of Aeronautics and Astronautics put forward a load monitoring system based on strain gauge, which is used to analyze the fatigue wear of landing gear during landing. However, the installation area of landing gear is limited, and all kinds of airborne equipment contain a certain degree of electromagnetic interference, which affects the accuracy of electrical sensing monitoring [5]. Fiber Bragg grating(FBG) has the advantages of light weight, reusability and strong anti-electromagnetic interference ability, which can effectively solve the problems faced by traditional strain monitoring [6,7,8,9]. It has been widely applied in various fields. For instance, Zhang et al. utilized fiber optic sensors to conduct freeze–thaw damage experiments on asphalt beams. Their results provided theoretical support for understanding the impact of freeze–thaw cycles on asphalt pavements and laid the foundation for the widespread application of distributed fiber optic sensors in evaluating structural performance under freeze–thaw conditions [10]. Smailov et al. achieved the measurement of high-frequency vibrations or pulsed strains (exceeding ±1000 με) through numerical analysis of FBG reflected power, and the feasibility of the method was verified via simulations and experiments [11]. Harasim proposed an interference-resistant bending measurement method based on tilted fiber Bragg grating(TFBG), enabling curvature detection by analyzing the quasi-linear changes in sensitive regions of the transmission spectrum. The method was validated for bending radii ranging from 30 to 12 mm [12]. Current research on FBG is also quite in-depth. For example, Kisała et al. revealed why only the spectral feature minima corresponding to certain cladding modes deform during TFBG bending. This finding holds significant implications for the application of Bragg gratings as bending sensors [13]. 

FBG sensing technology, as a mature optical fiber sensing method, has been widely applied in various fields such as civil engineering, energy, and aerospace. However, the mechanical characteristics, service environments, and monitoring requirements of different engineering structures exhibit significant differences. Therefore, research targeting specific structures is not merely a straightforward reuse of technology but rather an engineering-oriented expansion and innovation based on a unified theoretical framework. For these reasons, researchers worldwide have conducted extensive studies on the application of FBG in aircraft landing gear testing, as detailed below. 

In 2014, Korean scholar J.H. Kim proposed a light aircraft structure monitoring system based on FBG sensing. The load calibration coefficient was obtained through ground calibration test, and the measured strain was successfully matched with the historical flight load data [14,15].

In 2016, G. Petrone et al. from Italy developed a Health Monitoring and Management System(HMMS) for the landing gear of military trainers. They collected data using a multi-sensor network integrating Micro-Electro-Mechanical System (MEMS) accelerometers and FBG sensors placed at the maximum stress position of the landing gear. The team employed a specific algorithm for data signal processing and health state estimation [16]. In 2018, A. Iele et al. integrated optical FBG sensors into aircraft landing gear for remote and real-time load monitoring. They conducted load tests in a laboratory environment, and the results closely matched those from reference electric strain gauges. This demonstrated the significant potential of FBG sensing technology for remote and real-time load measurement in aircraft landing gear [17]. In 2022, A. Brindisi et al. deployed an FBG sensing array on a 1:3 scale spring landing gear model to monitor impact processes at different heights. The results showed that the FBG sensing system could detect impacts and the moment of landing gear lift-off with a resolution of 1 millisecond [18]. In 2025, Angela Brindisi et al. integrated a FBG network onto leaf spring landing gears to record the structural response under operational loads. This system was designed to detect hard landing conditions on soils with different absorption characteristics and to differentiate between soil types during landings. The results indicated that for multiple drops from the same height on the same soil, the cross-correlation coefficient of the sensor network measurements was very high, confirming the system’s high reliability in detecting landings [19].

M. Viscardi and others in the United States applied strain gauges and FBG and other non-contact monitoring methods to the main landing gear cabin of CS-25 aircraft, which proved the great potential of FBG sensing technology for remote and real-time load measurement of aircraft landing gear [20].

At present, the related work of structural testing of landing gear using optical fiber sensing technology generally focuses on a specific part of landing gear, such as the buffer, main strut and diagonal strut, and most of them are analyzed for specific loads, but there is a lack of research on the multi-directional loading of the overall structure of landing gear. Therefore, this study takes the main landing gear of a civil aircraft as the research object. To address the complex three-dimensional load monitoring requirements of aircraft main landing gears, an optimized FBG sensor deployment strategy and data regression analysis method are designed by considering their unique structural configuration, load-bearing characteristics, and installation environment. Furthermore, a load monitoring system tailored for this specific application scenario is developed.

## 2. Principle of FBG Strain Sensing and Load Monitoring

### 2.1. Principle of FBG Strain Sensor

**Figure 1:**
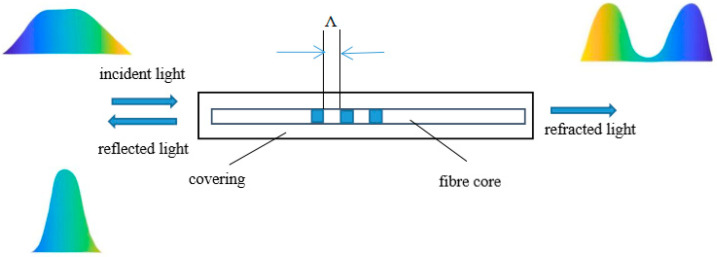
FBG sensing principle.

FBG sensor is a typical wavelength modulation sensor, which is mainly etched by excimer laser or ultraviolet exposure. When the broadband light source reaches the gate region, the light meeting the specific wavelength is reflected, while the light not meeting the specific wavelength is transmitted, and the resulting reflection spectrum presents a peak centered on Bragg wavelength, which is called Bragg wavelength [21]. The Bragg wavelength is the central peak wavelength in the FBG reflection spectrum, representing the specific wavelength at which coherent reflection occurs. The FBG sensing principle is shown in Figure 1.

Equation (1) describes the relationship between the Bragg wavelength, effective refractive index, and grating period.

(1) $$\lambda_{B}=2n_{eff}\Lambda$$

In Equation (1), $\lambda_{B}$ represents the Bragg wavelength, $n_{eff}$ is the effective refractive index of the fiber core, and Λ denotes the grating period length. When FBG is affected by external physical quantities, $\lambda_{B}$ will drift [22]. The variation value of Bragg wavelength can be obtained by differentiating Equation (1):

(2) $$\Delta \lambda_{B}=2\left[\Lambda \frac{\partial n_{eff}}{\partial l}+n_{eff}\frac{\partial \Lambda }{\partial l}\right]\Delta l+2\left[\Lambda \frac{\partial n_{eff}}{\partial T}+n_{eff}\frac{\partial \Lambda }{\partial T}\right]\Delta T$$

where $\frac{\partial n_{eff}}{\partial l}$ represents the change in effective refractive index of grating caused by strain, which is called elasto-optic effect; $\frac{\partial \Lambda }{\partial l}$ represents the expansion and contraction of grating period caused by strain; $\frac{\partial n_{eff}}{\partial T}$ indicates the change in effective refractive index of grating caused by temperature, which is called thermo-optic effect; $\frac{\partial \Lambda }{\partial T}$ represents the thermal expansion effect of grating period caused by temperature. When the control temperature is constant or the FBG strain sensor is decoupled, $\lambda_{B}$ can be expressed as Equation (3):

(3) $$\frac{\Delta \lambda_{B}}{\lambda_{B}}=(1-p_{e})\varepsilon$$

The effective photoelastic coefficient $p_{e}$ can be expressed as:

(4) $$p_{e}=\frac{n_{neff}^{2}}{2}\left[p_{12}-v(p_{11}+p_{12})\right]$$

where $p_{11}$ and $p_{12}$ represent the photoelastic coefficients of the optical fiber material, describing how mechanical strain affects its refractive index; v representing the ratio of transverse contraction to axial elongation under strain.

### 2.2. Monitoring Principle of Landing Gear Strain Load

Load monitoring is one of the core principles of th health monitoring of landing gear structure and an important way to predict fatigue life. Based on the characteristics that FBG sensors are easy to be networked, it is decided to establish strain–load matrix equations by using multiple linear regression algorithm, and realize the purpose of monitoring the three-dimensional load of landing gear through FBG strain sensors [23].

Taking the three-dimensional load on the landing gear as the independent variable of the system input and the strain measured by each FBG sensor as the dependent variable, the linear equations as shown in Formula (5) can be obtained [23]. In Formula (5), $P_{x}$ represents the heading load, $P_{y}$ represents the vertical loading, $P_{z}$ denotes the lateral loading, and $\varepsilon_{n}$ is the strain value of the sensor.

(5) $$\left\{\begin{array}{c} \varepsilon_{1}=H_{1x}P_{x}+H_{1y}P_{y}+H_{1z}P_{z}\\ \varepsilon_{2}=H_{2x}P_{x}+H_{2y}P_{y}+H_{2z}P_{z}\\ \vdots \\ \varepsilon_{n}=H_{nx}P_{x}+H_{ny}P_{y}+H_{nz}P_{z} \end{array}\right.$$

Formula (5) can be rewritten as a matrix equation as shown in Formula (6).

(6) $$\left[\begin{array}{c} \varepsilon_{1}\\ \varepsilon_{2}\\ \vdots \\ \varepsilon_{n} \end{array}\right]=\left[\begin{array}{ccc} H_{1x} & H_{1y} & H_{1z}\\ H_{2x} & H_{2y} & H_{2z}\\ \vdots & \vdots & \vdots \\ H_{nx} & H_{ny} & H_{nz} \end{array}\right]\left[\begin{array}{c} P_{x}\\ P_{y}\\ P_{z} \end{array}\right]$$

Formula (6) can be abbreviated as ε=HP, where ε and P are both known quantities, and ε is the column vector ${\left[\varepsilon_{1},\varepsilon_{2},\ldots ,\varepsilon_{n}\right]}^{T}$, representing the measured strains from FBG sensors installed on the landing gear. P is the column vector ${\left[P_{x},P_{y},P_{z}\right]}^{T}$, denoting the load components applied to the landing gear in the heading, vertical, and lateral directions, respectively. The coefficient matrix H can be calculated by the least square fitting from the strain data of the landing gear under unidirectional load only. The calculation method is shown in Formula (7) [23].

(7) $$H={\left[P^{T}P\right]}^{-1}P^{T}\varepsilon$$

After calculating the load-strain response coefficient matrix H, the load calibration coefficient matrix $\hat{H}$ can be obtained by transformating Formula (8) on the premise of ensuring linear independence among strain parameters.

(8) $$\hat{H}={\left[H^{T}H\right]}^{-1}H^{T}$$

Finally, by establishing the strain–load matrix equations as shown in Formula (9), a strain–load monitoring system with sensor strain data as input and landing gear three-dimensional load estimation as output can be established [23].

(9) $$\hat{P}=\hat{H}\varepsilon$$

$\hat{P}$ is the estimated load column vector ${{[\hat{P}}_{x},\;{\hat{P}}_{y},\;{\hat{P}}_{z}]}^{T}$, where ${\hat{P}}_{x}$, ${\hat{P}}_{y}$ and ${\hat{P}}_{z}$ represent the estimated load components in the heading, vertical, and lateral directions of the landing gear, respectively.

## 3. Simulation Analysis of Static Load of Main Landing Gear

### 3.1. Load Analysis of Main Landing Gear

The landing gear bears loads during the ejection and landing phases of the aircraft, and its ejection and take-off process can be divided into four stages: preparation stage, ejection slide, free slide and off-ground flight. In the preparation stage, the traction rod is connected with the front landing gear to accelerate the traction of the fuselage, and then the ejection rod is tensioned; After the ejection begins, the catapult continues to be loaded. When the release load is reached, the constant tension bolt is disconnected, and the aircraft is out of the constraint of the pin rod, and the initial speed meets the requirements, and it is ready to fly off the ship for free sliding [24]. During takeoff, the main landing gear mainly bears the supporting reaction and friction of the deck. During the landing of the aircraft, the main landing gear first lands on the deck rapidly, and after sliding for a certain distance, the arresting cable pulls the arresting hook, and the speed of the aircraft decreases rapidly until it stops on the deck safely. The main landing gear of carrier aircraft mainly bears the impact load, blocking load and friction forces during landing [25,26].

**Figure 2:**
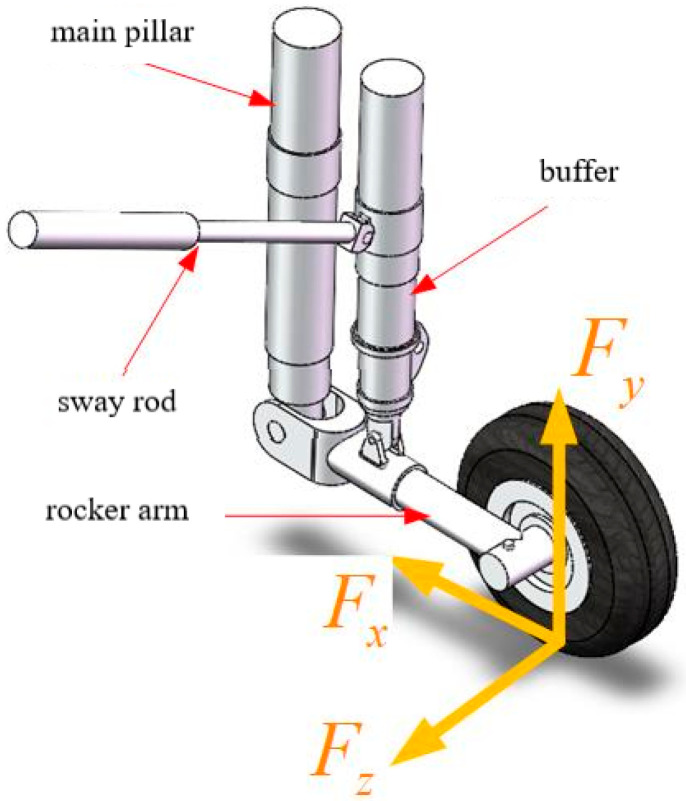
Schematic diagram of main landing gear structure.

Taking the reverse heading as the X axis, the direction perpendicular to the ground as the Y axis, and the section perpendicular to the hub as the Z axis, a space rectangular coordinate system is established. In order to facilitate the study, the complex space loads borne by the main landing gear in the stages of catapult takeoff, landing and deck operation can be simplified and decomposed into heading load $F_{x}$, vertical load $F_{y}$ and lateral load $F_{z}$, as shown in Figure 2. The three-dimensional load on the ground is transmitted to the lower rocker arm of the main landing gear through the wheel, and then transmitted to the fuselage through the buffer, main strut, diagonal strut and other structures. Because of the buffering effect of the hydraulic structure of the strut, the large ground three-dimensional load generated when the main landing gear takes off or lands is greatly reduced when it is transmitted to the fuselage through the buffering strut, so the response of the rocker arm structure directly connected with the wheel can best reflect the real ground three-dimensional load, and the relative position from the point where the wheel load acts to the rocker arm does not change with the stroke of the buffer.

### 3.2. Simulation Analysis of Landing Gear Strain Response

**Figure 3:**
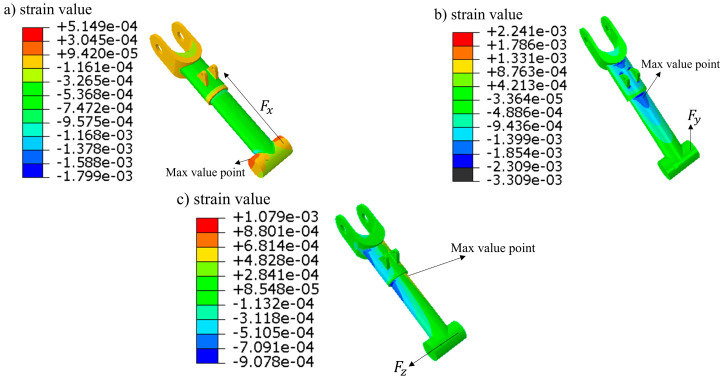
Finite element simulation strain nephogram of landing gear rocker arm. (**a**) heading loading (**b**) vertical loading (**c**) lateral loading.

**Figure 4:**
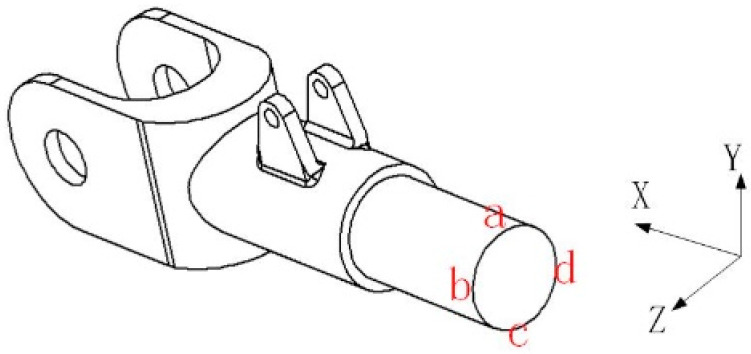
Schematic diagram of cross-sectional position of rocker arm.

The main landing gear rocker arm directly bears and transmits the three-dimensional loads acting on the dummy wheel structure during aircraft landing, making it the selected object for experimental monitoring. To determine the appropriate locations for sensor installation, a three-dimensional model of the rocker arm structure was established based on the actual loads experienced during the landing gear’s operation. The connection between the rocker arm structure and the shock absorber was fixed as a constraint, and the following loads were applied: a heading load $F_{x}$ of 24 kN, a vertical load $F_{y}$ of 69 kN, and a lateral load $F_{z}$ of 8 kN. The axial strain distribution is shown in Figure 3. To facilitate the description of the strain response state at various points on the rocker arm, Figure 4 presents a schematic cross-section of the rocker arm. A finite element static simulation of the model was performed using Abaqus 2022, with the solver type set to an explicit solver. The rocker arm is manufactured from aviation steel, with material properties including an elastic modulus of 200 GPa, a Poisson’s ratio of 0.29, and a density of 7.85 g/cm^3^. After defining the material properties in Abaqus, the model was meshed using a three-dimensional stress mesh with tetrahedral elements and a size of 1.4.

As can be seen from the above simulation cloud picture, when the rocker arm structure is subjected to the heading load $F_{x}$ (Figure 3a), the compression occurs at ‘a’, ‘b’, ‘c’ and ‘d’ of its cross-section. When the rocker arm structure is subjected to vertical load $F_{y}$ (Figure 3b), the position ‘a’ of its circular shaft section is compressed, and the strain is about −488.6 με, and the corresponding position ‘c’ is stretched, and the strain is about 501 με. The positions ‘b’ and ‘d’ are equivalent to the neutral layer of the structure under bending moment, and the strain is close to zero. When the rocker arm structure is subjected to lateral load $F_{z}$ (Figure 3c), the point ‘b’ of the rocker arm structure is compressed, and the strain is about −311.8 με; the corresponding point ‘d’ is stretched, and the strain is about 324.1 με. The point ‘a’ and ‘b’ are equivalent to the neutral layer of the structure when subjected to bending moment, and the strain is close to zero.

The simulation results show the strain response law and trend of each structure under different loads, which can provide reference for the subsequent sensor placement and networking scheme design.

## 4. Scheme Design of FBG Sensor Networking

**Figure 5:**
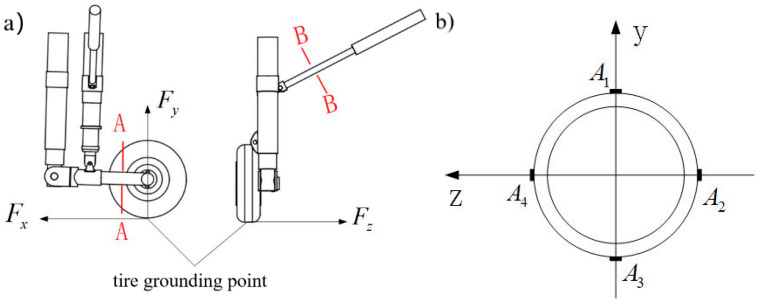
Location map of strain sensor. (**a**) selection of monitoring section (**b**) installation position of strain sensor on section A.

During the take-off and landing of aircraft, the rocker arm structure on the main landing gear directly bears the three-dimensional load, and there is enough space for installing FBG sensors on the rocker arm structure, so the rocker arm section is selected as the strain monitoring position. Before employing FBG sensors in this study, a comprehensive analysis of their metrological characteristics was conducted. Through standardized loading experiments and temperature-controlled tests, key performance indicators such as sensitivity, linearity, repeatability, and temperature coefficient were evaluated. The measurement results were quantitatively assessed with uncertainty analysis, thereby validating the sensors’ reliability and precision. The FBG sensors, featuring a strain resolution of 1 με and a strain sensitivity of 1.18 pm/με, were selected to ensure precise strain acquisition. According to the axial strain distribution on the rocker arm structure, FBG sensors are arranged in the area near the buffer on the rocker arm. As shown in Figure 5, four FBG strain sensors, $A_{1}$, $A_{2}$, $A_{3}$ and $A_{4}$, which are distributed in a circumferential array, are stuck along the axial direction at the cross-section from the total length L/4 of the rocker arm. The FBG sensor adopts a substrate-based pre-stretched packaging method and is bonded to the structure under test using epoxy adhesive. The strain measurement model is shown in Figure 5a. This packaging approach ensures consistency and results in a more stable strain transfer coefficient. According to experience, when the main landing gear is subjected to lateral load, the strain response on the diagonal strut is the largest, so a sensor, numbered $B_{1}$, is arranged on the section B of the diagonal strut near the actuator in the axial direction to monitor the lateral load.

**Figure 6:**
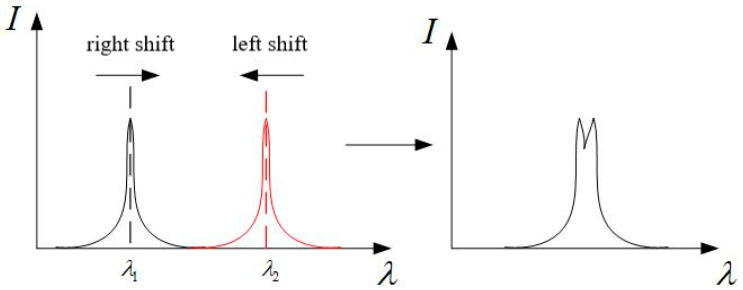
Phenomenon central wavelength coincidence.

Four sensors on section A are welded together to form line 1, which is quasi-distributed strain sensing. At this time, the initial center wavelength spacing of four FBG sensors needs to be considered. If the wavelength spacing between adjacent sensors is too small, when the center wavelength shifts, the superposition of two peaks will lead to the failure of Fung Cham algorithm(the peak detection algorithm integrated within the demodulation instrument), resulting in erroneous strain data, as shown in Figure 6. Figure 6 demonstrates the spectral overlap phenomenon that occurs when the initial central wavelength spacing between adjacent FBG sensors is too small and wavelength shifts occur. In the left diagram, the reflection peak corresponding to $\lambda_{1}$ shifts to the right, while the peak corresponding to $\lambda_{2}$ shifts to the left, resulting in an overlap between their peak regions. The right diagram shows the morphology of the superimposed spectrum: the originally distinct two independent peaks merge into a distorted spectral profile with a bimodal structure. Such spectral distortion can cause the Fung Cham peak detection algorithm embedded in the demodulator to fail in accurately identifying the peak positions, thereby leading to erroneous strain data interpretation.

The wavelength gap between FBG is set to be greater than 6nm. According to the sensitivity (1.2 pm/με) of the packaged FBG strain sensor, the strain interval range of adjacent sensors is calculated to be 5000 με, which meets the measurement requirements. Four FBG strain sensors with initial center wavelengths of 1531 nm, 1542 nm, 1552 nm and 1561 nm were selected and connected to an optical fiber by a welding machine. Among them, there is a large strain at the position where the sensor $A_{1}$ is attached; thus, a large wavelength shift range is observed on both sides. The center wavelength of the sensor on section B is 1551 nm, and it is connected to the demodulator through line 2.

## 5. Calibration and Regression Analysis of Ground Load of Main Landing Gear

### 5.1. Landing Gear Fixing and Loading Mode

The ground calibration test of landing gear load was carried out in a factory building. The main strut, bumper, diagonal strut and other structures of the main landing gear are upside down and fixed on the bench fixture connected with the wing. The external load acts on the dummy wheel structure installed on the main landing gear through the loading actuator and the steel cable, and the loading actuator is fixed on the triangular bracket, and the load is controlled through the matching hydraulic system; A force sensor is installed between the cable and the dummy wheel to measure the real load applied to the landing gear structure.

**Figure 7:**
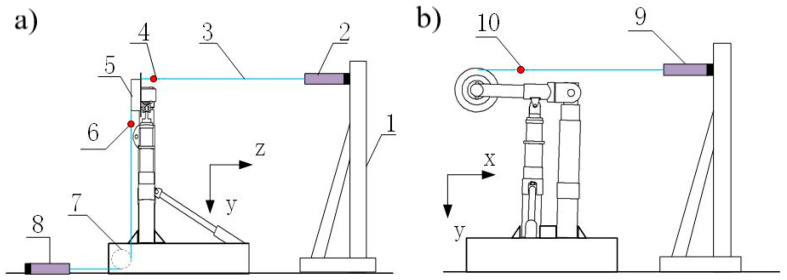
Schematic diagram of main landing gear loading form. (**a**) schematic diagram in ZOY plane (**b**) schematic diagram in XOY plane.

Figure 7 presents a simplified 2D model of the loading system, with component numbering specifications provided in Table 1. During loading operations, the hinge mechanism of the diagonal strut becomes locked, maintaining a fixed angular position relative to the Y-axis. The system employs three actuators (numbered 2, 8, and 9) to apply lateral, vertical, and heading loads, respectively. Corresponding force measurements are obtained through sensors numbered 4 (lateral load), 6 (vertical load), and 10 (heading load), which monitor the forces exerted on the dummy wheel structure. Notably, actuator no. 8 incorporates a pulley mechanism to redirect the applied load into the vertical direction.

### 5.2. Ground Calibration Load Loading Scheme

**Figure 8:**
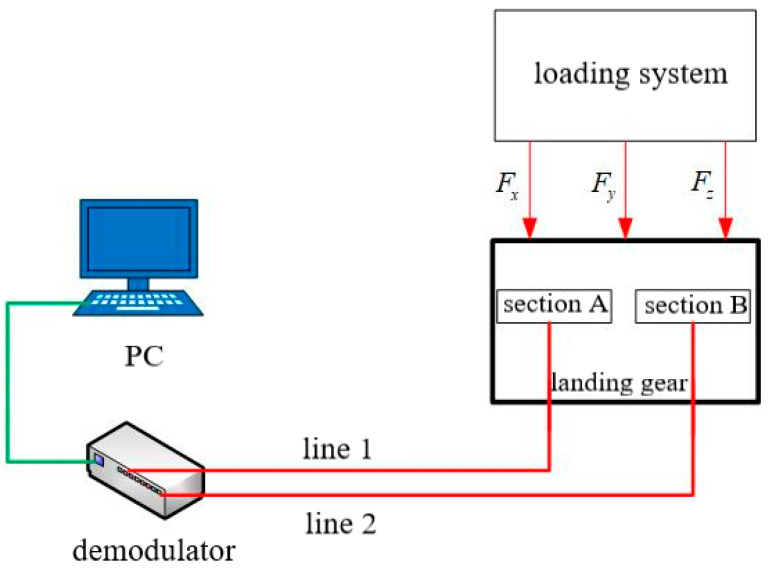
Schematic diagram of ground calibration system.

The load loading system applies a three-dimensional load to the landing gear, and the FBG sensors installed on sections A and B are subjected to strain, so that the central wavelengths of the sensors shift. Optical signals enter the demodulator through optical fibers, and the wavelength signals are converted into digital signals and input to the PC. The central wavelength data of each FBG sensor is obtained through the algorithm program on the PC, and converted into strain data according to the measured sensitivity. The schematic diagram of the landing gear load calibration test system is shown in Figure 8. Line 1 and line 2 in Figure 8 correspond to the two optical fiber lines mentioned in Section 4, which are connected to two separate channels of the demodulator, respectively.The sampling frequency of the demodulator is 100Hz.

After the test system is built, the load loading condition is designed. According to the superposition theorem of material mechanics, the strain produced on the measuring section of landing gear can be regarded as a linear superposition of the results of the combined action of loads applied to the landing gear wheel, and its expression is as follows:

(10) $$\varepsilon =k_{x}F_{x}+k_{y}F_{y}+k_{z}F_{z}$$

where the k is strain response coefficient under the corresponding load. Therefore, when $F_{y}=F_{z}=0$, the response coefficient $k_{x}$ of heading load $F_{x}$ can be measured by single heading loading; Similarly, by controlling $F_{x}=F_{z}=0$, the strain response coefficient of vertical load $F_{y}$ can be measured through a single vertical loading; By controlling $F_{x}=F_{y}=0$, the strain response coefficient of lateral load $F_{z}$ is measured by unilateral loading. Based on this, three unidirectional loading conditions are designed, the maximum value of unidirectional loading is $F_{max}$, and the single-stage increasing load is $F_{max}/10$. According to the maximum limit load of heading load ($F_{x}$) 24 kN, vertical load ($F_{y}$) 69 kN and lateral load ($F_{z}$) 8 kN, the load value of 10 grades is designed in equal proportion, and it will increase step by step when loading.

For the measurement of three-dimensional load in space, the calibration of one-dimensional load is not enough, and it is necessary to design two-dimensional and three-dimensional combined loading tests to characterize the bearing state of carrier-based aircraft landing gear in working state. See Table 2 for the loading conditions of each working condition. 

### 5.3. Establishment and Teste of Strain–Load Matrix Equation

When the motor applies a load to the landing gear, the strain generated in the structure is transmitted to the sensor at this point, which further causes the reflection spectrum shift in the FBG. The central wavelength is extracted and recorded from the reflection spectrum in real time by interpolation of the Fung Cham algorithm. After temperature decoupling, the central wavelength drift of the strain sensor caused by temperature change is removed. The difference between the average wavelength data and the initial wavelength when each load is stable is taken as the FBG wavelength change under this load, and the corresponding strain value can be obtained by combining the strain sensitivity.

**Figure 9:**
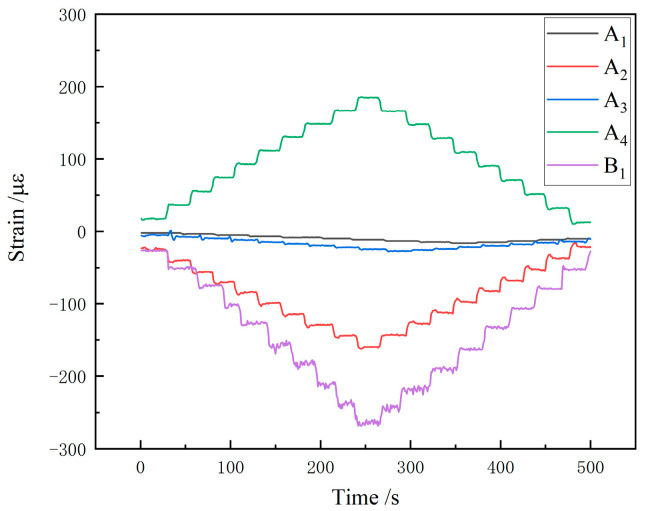
Strain response curve of lateral single loading.

**Figure 10:**
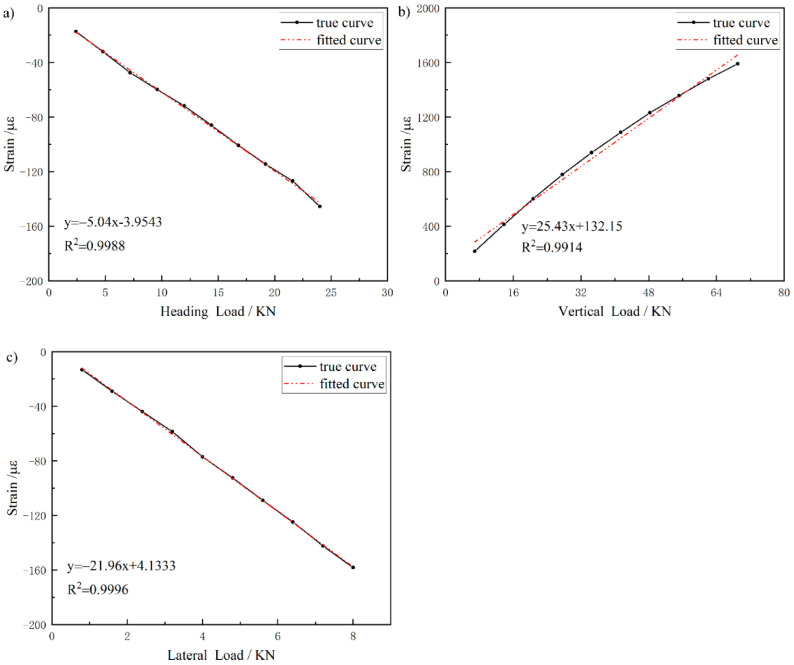
One-way load fitting result of sensor $A_{2}$.

**Figure 11:**
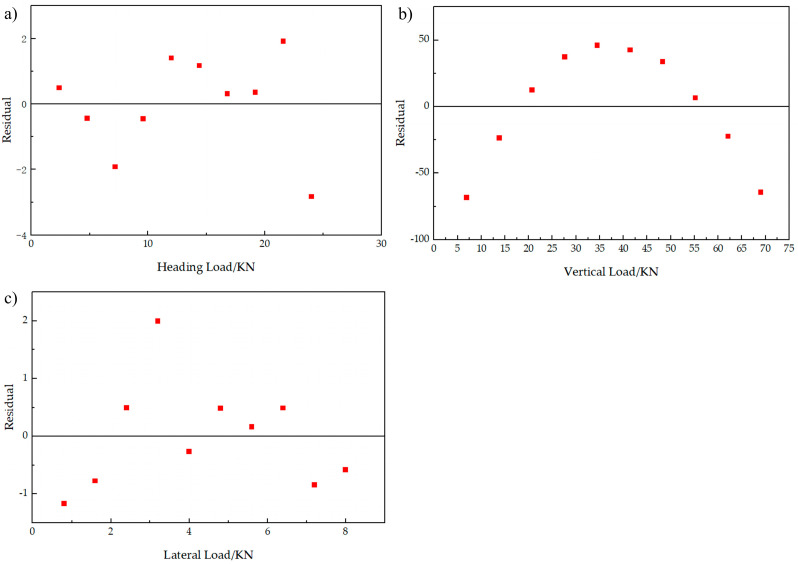
Residual plot for the regression analysis in Figure 10.

Figure 9 is the strain acquisition curve of five measuring points under the condition of lateral single loading, which shows that when lateral single loading is applied, measuring point $A_{2}$ and $B_{1}$ is compressed, the measuring points $A_{4}$ are stretched, and the measuring points $A_{1}$ and $A_{3}$ are located below the neutral axis of the rocker arm, then they are both compressed, which is consistent with the simulation trend. The least square fitting is carried out between each level of load and the corresponding strain value in the unidirectional loading condition. Taking sensor $A_{2}$ as an example, the fitting results of its three unidirectional loading fitting results are shown in Figure 10a–c. Figure 10a presents the fitted curve under the heading single-sided loading condition. The black solid line represents the measured strain curve (true curve), while the red dashed line denotes the least-squares fitted curve (fitted curve). The strain exhibits a clear linear negative correlation with the heading load, with a fitting equation of y=−5.04x−3.9543 and a coefficient of determination $R^{2}=0.9988$, indicating exceptionally high fitting accuracy. Figure 10b shows the fitted curve under the vertical single-sided loading condition. In this case, the strain demonstrates an approximately linear positive correlation with the vertical load. The fitting equation is y=25.43x+132.15, with $R^{2}=0.9914$, reflecting a strong fitting performance. Figure 10c illustrates the fitted curve under the lateral single-sided loading condition. Here, the strain displays an approximately linear negative correlation with the lateral load. The fitting equation is y=−21.96x+4.1333, with $R^{2}=0.9996,$ again indicating an excellent level of fitting accuracy. The residual plot for the regression analysis in Figure 10 is shown in Figure 11.

.

According to the calculation method of measuring point $A_{2}$, strain load response coefficients $K_{x}$, $K_{y}$ and $K_{z}$ of five measuring points under three unidirectional loads can be calibrated, respectively, and the results are shown in Table 3.

As can be seen from Table 3, there is no linear correlation between the coefficient vectors of the five measuring points, and the number of independent strains is greater than the load parameters, so $\hat{K}$ can be estimated by inverting the coefficient matrix K in matrix Formula (8). The strain-load prediction equation under this system is calculated as follows

(11) $$\hat{F}=\left[\begin{array}{c} {\hat{F}}_{x}\\ \hat{F_{y}}\\ \hat{F_{z}} \end{array}\right]=\left[\begin{array}{ccccc} 0.1199 & 0.0484 & 0.0516 & 0.0184 & -0.0324\\ -0.0171 & 0.0055 & 0.0067 & 0.0077 & 0.0018\\ -0.0499 & -0.0035 & -0.0219 & -0.0052 & -0.0031 \end{array}\right]\left[\begin{array}{c} \varepsilon_{1}\\ \varepsilon_{2}\\ \varepsilon_{3}\\ \varepsilon_{4}\\ \varepsilon_{5} \end{array}\right]$$

In order to verify the accuracy of the load prediction equation, the strain values obtained under biaxial and triaxial loading conditions as verification conditions are substituted into the equation. The prediction results and relative errors are shown in Table 4.

It can be seen from Table 4 that the maximum relative error between the calculated three-dimensional landing gear load and the actually applied load is 4.00% under the bidirectional loading verification condition, which appears in the heading loading of Condition 6; Under the three-dimensional load verification condition, the maximum relative error between the calculated three-dimensional load of landing gear and the actually applied load is 2.35%, which appears in the vertical load. The average relative errors of heading load, vertical load and lateral load are 2.90%, 1.14% and 2.78%, respectively. Generally speaking, the absolute values of the relative errors between the three-dimensional load calculated by the strain–load matrix equation and the actual load are all within 4%, which meets the engineering standards and proves the feasibility of the proposed load real-time monitoring method.

It is considered that the error of back calculation load is mainly caused by the deviation between the load indication applied by the loading test bench and the real load size, and the other is the error that may exist in the process of calculating the center wavelength by Fung Cham interpolation of the reflection spectrum by the demodulation system and the error introduced in the process of calculating the strain–load equation.

## 6. Conclusions

In this paper, the main landing gear of an aircraft is taken as the research object. By building a sensing networking system with FBG strain sensor as the core, the three-dimensional load calibration test is carried out on the ground, and the strain response of the rocker arm and diagonal strut structure is monitored and collected online in real time, and the strain–load prediction model for this system is established. The feasibility of this model is verified under the working conditions of two-way combined loading and three-way combined loading, which provides a reference for the engineering practice of FBG in the field of aircraft structural load monitoring. In the follow-up study, it is planned to extend the monitoring object to other aircraft structures, introduce impact testing to simulate the real landing gear landing state, and explore the fatigue law of the structure caused by impact loads.

Under the measurement conditions in this study, although the correspondence between load and strain was obtained, the load at this stage was applied through ground loading. For the landing gear, during actual dynamic flight, factors such as the magnitude, direction, and form of the loads it bears, as well as vibrations during the flight process, will inevitably interfere with the collected data. Whether ground calibration alone can simulate these conditions remains to be further investigated, and determining how to eliminate the interference from these factors should be a potential direction for future research.

## Figures and Tables

**Table 1:** List of main landing gear loading system.

Serial Number	Structure Name	Serial Number	Structure Name
1	triangle support	6	$\mathrm{force}\;\mathrm{sensor}\;(F_{y}$)
2	$\mathrm{actuator}\;\mathrm{cylinder}\;(F_{z}$)	7	pulley
3	cable wire	8	$\mathrm{actuator}\;\mathrm{cylinder}\;(F_{y}$)
4	$\mathrm{force}\;\mathrm{sensor}\;(F_{z}$)	9	$\mathrm{actuator}\;\mathrm{cylinder}\;(F_{x}$)
5	Undercarriage wheel	10	$\mathrm{force}\;\mathrm{sensor}\;(F_{x}$)

**Table 2:** Loading table of ground calibration test conditions.

Test Conditions	Heading Load/KN	Vertical Load/KN	Lateral Load/KN
1	24	0	0
2	0	69	0
3	0	0	8
4	10	42	0
5	0	50	8
6	10	0	8
7	15	49	6

**Table 3:** Strain load coefficient of each FBG measuring point.

Measuring Point	$K_{x}/\mu \varepsilon \cdot N^{-1}$	$K_{y}/\mu \varepsilon \cdot N^{-1}$	$K_{z}/\mu \varepsilon \cdot N^{-1}$
$A_{1}$	3.5746	−27.2328	−1.0280
$A_{2}$	−5.0412	25.4346	−21.9562
$A_{3}$	3.9870	29.5193	−1.02830
$A_{4}$	11.0506	26.1761	20.558
$B_{1}$	−12.5477	−0.9178	−26.5656

**Table 4:** Load prediction and error under verification condition.

Verification Conditions		Actual Load/KN	Predicted Load/KN	Relative Error/%
Condition 4	$F_{x}$	10	9.61	3.90
	$F_{y}$	42	41.63	0.88
	$F_{z}$	0	0.87	/
Condition 5	$F_{x}$	0	-0.31	/
	$F_{y}$	50	50.54	1.08
	$F_{z}$	8	8.27	3.38
Condition 6	$F_{x}$	10	9.6	4.00
	$F_{y}$	0	0.03	/
	$F_{z}$	8	7.75	3.13
Condition 7	$F_{x}$	15	14.88	0.80
	$F_{y}$	49	47.85	2.35
	$F_{z}$	6	6.11	1.83

## Data Availability

Restrictions apply to the datasets. The dataset presented in this article is not readily available as the data are part of an ongoing follow-up study. For requests to access the dataset, please contact email: yanguang79@bistu.edu.cn.
